# A Case of Acquired Angioedema Leading to the Diagnosis of Systemic Lupus Erythematosus

**DOI:** 10.7759/cureus.50054

**Published:** 2023-12-06

**Authors:** Prakash Shende, Avani Reddy, Vikram B Vikhe, Ahsan A Faruqi, Devansh Khandol

**Affiliations:** 1 General Medicine, Dr. D. Y. Patil Medical College, Hospital and Research Centre, Pune, IND

**Keywords:** lymphoproliferative disorder (lpd), c1 esterase inhibitor, hypocomplementemia, acquired angioedema (aae), systemic lupus erythematosus (sle)

## Abstract

We describe a case of a young 32-year-old Indian female who presented with a solitary symptom of facial swelling for two months. The patient’s blood test results showed hypocomplementemia and C1 INH deficiency and fell into the “third type” of acquired angioedema (AAE), leading to the diagnosis of systemic lupus erythematosus (SLE), with SLE inactivity at the time of presentation, which makes this an interesting case due to the rarity of such findings in our clinical settings.

## Introduction

Angioneurotic edema is defined as edema that is localized and affects the subcutaneous tissue and deeper layers of the skin. Angioedema can be acquired, or it can run in the family. The symptoms of acquired angioedema (AAE) are caused by bradykinin secreted because of incorrect activation of the contact-kinin system, an acquired deficit of C1 inhibitor (C1 INH), and hyperactivation of the classical route of human complement. Reports of acquired angioneurotic edema secondary to systemic lupus erythematosus (SLE) are uncommon [1].

For AAE, two primary mechanisms are understood. Type 1 is defined by the rapid catabolism of the C1 esterase inhibitor, and type 2 is distinguished by the presence of an autoantibody against the enzyme. In brief, two possible mechanisms cause AAE that are associated with lymphoproliferative disorder (LPD) [2]. Our case report outlines an uncommon situation of facial angioedema without LPD but secondary to SLE.

## Case presentation

A 32-year-old Indian Maharashtrian female with no known co-morbidities presented to us with complaints of facial swelling for two months, which was not associated with any itching or discoloration of the skin. She had no history of consumption of any drugs, uncommon meals, insect bites, or other factors prior to the onset of facial swelling. She had no history of fever, myalgia, decreased urine output, rash, chest pain, breathlessness, weight loss, or trauma. She also had no history of oral ulcers, dental infections, alopecia, or arthralgia. She had regular monthly menstrual cycles. She had no history of similar complaints in the past and an unremarkable family history.

On examination, blood pressure was 100/70 mmHg, pulse rate was 80/min, and oxygen saturation was 98% on room air. A local examination of her face showed soft, painless swelling on the bilateral buccal area, along with swelling of the lips. A systemic examination revealed no abnormalities. Chest X-ray, electrocardiography, and USG abdomen and pelvis reports were within normal limits. Routine blood investigations along with complement levels were sent to further evaluate the patient (Tables 1-2).

**Table 1:** Blood reports on Day 1 of admission SGOT: serum glutamic oxaloacetic transaminase, SGPT: serum glutamic pyruvic transaminase, ALP: alkaline phosphate, TSH: thyroid-stimulating hormone, CRP: C-reactive protein, ESR: erythrocyte sedimentation rate, IgE: immunoglobulin E

Blood tests	Result	Reference range
Haemoglobin	11.30 g/dl	12.3-15.3 g/dl
Total leucocyte count	9,300/ul	4000-10000/ul
Platelet count	180,000/ul	150000-400000/ul
Eosinophils	1%	1-6%
Serum urea	17 mg/dl	17-49 mg/dl
Serum creatinine	0.72 mg/dl	0.6-1.2 mg/dl
Serum total bilirubin	0.56 mg/dl	0.10-1.20 mg/dl
Conjugated bilirubin	0.22 mg/dl	0.2-0.3 mg/dl
Unconjugated bilirubin	0.34 mg/dl	0.1-1.0 mg/dl
SGOT	14 U/Lt	8-43 U/Lt
SGPT	9 U/Lt	7-55 U/Lt
ALP	46 U/Lt	35-104 U/Lt
Total protein	7.0 g/dl	6.0-8.3 g/dl
Serum albumin	4.0 g/dl	3.4-5.0 g/dl
Serum globulin	3.0 g/dl	2.3-3.5 g/dl
Serum sodium	137 mmol/Lt	136-145 mmol/Lt
Serum potassium	4.60 mmol/Lt	3.50-5.10 mmol/Lt
Serum chloride	99.0 mmol/Lt	98-107 mmol/Lt
TSH	1.29 uIU/L	0.35-4.94 uIU/L
T3	0.67 ng/ml	0.64-1.52 ng/ml
T4	6.37 Ug/dl	4.87-11.72 ug/dl
CRP	1.2 mg/dl	0.8-1.0 mg/dl
ESR	75 mm/hr	20-30 mm/hr
Serum IgE	160 IU/ml	150-300 IU/ml
Urine routine microscopy	Within normal limits	

**Table 2:** Blood test results on Day 3 and Day 4 of admission ANA blot: antinuclear antibody blot test, C1 INH: C1 esterase inhibitor, ANA by IF: antinuclear antibodies detected by indirect immunofluorescence assay

Blood tests	Result	Reference range
Complement 3	28 mg/dl	88 to 201 mg/dl
Complement 4	2 mg/dl	15-45 mg/dl
C1 INH	0.006 g/dl	0.20-0.35 g/dl
Serum cortisol	6 mcg/dl	5-25 mcg/dl
ANA by IF	Positive (titer >1:160)	Significant titer >1:100
ANA blot	Positive for SS-A, dsDNA, and nucleosomes	

After noting the results, we evaluated them according to the SLE criteria. Although there was an absence of obvious clinical symptoms that we see in cases of SLE since ANA titers were elevated, along with hypocomplementemia and positive results for anti-dsDNA antibodies, we diagnosed her as AAE secondary to SLE, as the laboratory evidence was undeniable and criteria were met.

The patient was started on tablet methotrexate 15 mg (once a week), tablet hydroxychloroquine 200 mg (twice a day), tablet prednisolone 10 mg (twice a day), and tablet folic acid 5 mg (three times a week).

She was discharged five days after admission on prednisolone, hydroxychloroquine, methotrexate weekly, and folic acid. During her regular follow-ups, her steroid dose was tapered and eventually stopped, and methotrexate weekly, folic acid, and hydroxychloroquine were continued. These visits showed a reduction in facial swelling, with no presentation of new symptoms, and normalization of C3, C4, and C1 INH levels (90 mg/dl, 30 mg/dl, and 0.20 g/dl, respectively) with no further complications.

## Discussion

AAE in lupus is uncommon, and angioedema in SLE is predicted to have an incidence of less than 1%. Therefore, there should be a suspicion of non-allergic causes when a patient presents with only localized facial swelling, as in our case. Complement levels and C1 INH can help in diagnosis [2].

Anti-C1 INH antibodies or increased catabolism of C1 INH in combination with LPDs may be a contributing factor to kinin-mediated AAE. This can be distinguished from hereditary angioedema by its later age of onset, lack of family history, and low levels of C1q [3].

In SLE, a “third type” of AAE has been hypothesized to be connected to the low levels of the complements C3 and C4, transiently low C1 INH levels, and the absence of anti-C1 INH autoantibodies, and clinically, SLE is inactive during acute angioedema episodes [4].

In a recently conducted study, a total of 90,485 hospitalizations with an angioedema diagnosis were detected; of these cases, 1,505 had concurrent diagnoses of SLE and angioedema. Results showed that the most common type of patient with angioedema with SLE is usually young (mean age 44) and more likely to be female (89%) and African American (57%) [3]. In the same study, only 9% of the subjects were of Asian ethnicity, and cases associated with congenital enzyme deficiency (including C1 INH deficiency) were only 0.66%. These results also help to show us the rarity of our case.

According to a theory, the raised kinin levels in AAE could increase vascular permeability and make it easier for antibodies, cytokines, and chemokines to enter the cerebral circulation, raising the risk of CNS lupus [5]. Complications can be vast, such as pneumonia and sepsis; thus, early diagnosis in cases with unusual presentations and prevention of complications should be our mainstay of treatment.

The way the patient with AAE is approached should initially focus on minimizing fatalities like laryngeal edema from angioedema, followed by preventing the challenges brought on by recurrent angioedema. Laryngeal edema is a root cause of mortality from angioedema [6]. Immunosuppressive therapy for angioedema was connected to the results of levels of C3, C4, and C1 INH normalizing after treatment [4].

Newer treatment modalities show C1 INH concentrate therapy being used for acute attacks in AAE. Treatment with plasma-derived C1 INH concentrate consistently reduced the length of episodes of angioedema, as shown in a study of 44 patients with C1 INH-deficient AAE [7].

## Conclusions

Considering the various complications that can occur due to angioedema and the increased risk of mortality, solitary symptoms such as facial edema in a female patient must be evaluated thoroughly, regardless of ethnicity or age. As seen in this case, our patient improved significantly and was able to avoid recurrent angioedema and complications of SLE due to timely diagnosis and treatment. This case report describes a rare entity that should be part of our differential diagnosis when we approach a patient with similar complaints.
